# Public research funding and pharmaceutical prices: do Americans pay twice for drugs?

**DOI:** 10.12688/f1000research.24934.1

**Published:** 2020-07-15

**Authors:** Rena M. Conti, Frank S. David

**Affiliations:** 1 Questrom School of Business, Boston University, Boston, MA, 02215, USA; 2 Institute for Health System Innovation and Policy, Boston University, Boston, MA, 02215, USA; 3 Pharmagellan LLC, Milton, MA, 02186, USA; 4 Harvard-MIT Center for Regulatory Science, Boston, MA, 02115, USA

**Keywords:** Pharmaceutical, drug, price, access, research, development, funding, patents

## Abstract

In the debate over prescription drug pricing, some pharmaceutical industry critics claim that U.S. taxpayers pay twice for costly therapies, because publicly supported research is a major contributor to drug discovery and American taxpayers are inadequately rewarded for their research investment due to high drug prices. In fact, the empirical evidence supporting these claims is weak, and the pay twice argument distracts from important efforts to ensure that impactful new drugs continue to be developed and made widely available to patients who need them.

## Introduction

Do pharmaceutical companies unfairly bilk American patients when they charge exorbitant prices for drugs developed based on publicly funded research? In a hearing of the House Committee on Oversight and Reform in January, 2019, U.S. Representative Alexandria Ocasio-Cortez of New York argued that “the public is acting as early investor, putting tons of money into the development of drugs that then become privatized, and then they receive no return on the investment that they have made” (see video recording from Twitter).

Embedded in this “pay twice” argument are three key questions. First, to what extent is public funding responsible for the invention of most new drugs? Second, are U.S. taxpayers inadequately rewarded for their contribution to new drug development? And third, how are these claims salient to the national debate over drug prices?

In this piece, we summarize the available evidence regarding each of these questions as follows: First, although public funds support a significant amount of important biomedical research, the vast majority of the credit for translating this research into new therapies is due to private companies. Second, the contention that taxpayers receive “no return” on their research investment is incorrect, as it fails to account for the massive health and wealth benefits that Americans receive from new drugs. And finally, the pay twice claim distracts attention from far more impactful and feasible efforts to balance innovation and access across the entire pharmaceutical sector.

## Are new drugs invented by publicly funded researchers?

There is little debate that public funding of basic science is a critical enabler of drug development ^1, 2^. The National Institutes of Health (NIH) is the world’s largest government funder of biomedical research, and makes financial and practical contributions to all stages of it, including pre-clinical scientific investigations, translational medicine, and clinical trials. Detailed case studies reveal that public support has played at least some role in virtually all of the 26 most clinically and commercially significant drugs and drug classes approved over the past several decades ^3^. And several important medicines were solely invented by academic researchers, including the lung cancer therapy pemetrexed, the vitamin D analog doxercalciferol, the inhaled pulmonary vasodilator nitrous oxide, and the vaccine for Haemophilus influenzae type b ^4^.

But in a large majority of cases, the public sector’s contribution to new drugs has been in the form of early scientific findings, unrelated to current or potential applications. The public sector supported key basic research for 19 of the 26 “transformative” drugs and drug classes cited above, contributed to the actual discovery of a new therapy in just 11, and could claim sole discovery credit in only four cases ^5^. More broadly, although NIH funding supported at least one publication related to each of the 210 new medicines approved by the Food and Drug Administration (FDA) from 2010 to 2016, over 90 percent of those papers were related to the underlying drug target, not the actual therapy itself ^6^.

Comprehensive reviews of the genesis of approved drugs confirm that while publicly funded science often characterizes important pathologic processes and identifies potential drug targets, the private sector is the main inventor of most new therapies. A recent study found that for only 25 percent of drugs approved from 2008 to 2017 was there any documented contribution, of any magnitude, to a drug’s initial discovery, synthesis, or key intellectual property by a public sector research institution or academic “spin-off” company ^7^. This finding corroborated a review of approvals from 1998 to 2007, which found that publicly funded research helped either identify the chemical structure of the final compound or its direct antecedents or demonstrated therapeutic proof-of-concept for the target for only about a third of new drugs ^8^. If one uses a more stringent definition of “contribution” based solely on intellectual property, then taxpayers’ role in drug discovery is even smaller; less than 15 percent of new medicines are covered by even a single patent that was either directly issued to a public entity or contains a “government interest statement” acknowledging public funding ^7, 9, 10^.

Government funding makes enormous contributions to medicine by generating novel insights into biology and disease. But accumulated evidence demonstrates that in the majority of cases, it is the private sector, not academia, which translates those insights into new therapeutics.

## Are U.S. taxpayers inadequately rewarded for their investment in drug development?

A key component of the pay twice argument is that Americans receive an insufficient return from the funds they allot to biomedical research that enables new drug development. But although it is technically true that direct returns to the NIH from licensing royalties comprise a miniscule fraction of the agency’s budget, this strictly transactional assessment ignores the health and wealth benefits that accrue to taxpayers from publicly funded science.

In fact, the main return on investment American taxpayers expect from supporting biomedical research is in the form of direct benefits to morbidity and mortality – which have largely been realized. Therapies enabled by publicly funded science have extended and improved human lives, and enabled patients to avoid hospitalizations and other costly interventions that yield worse outcomes ^11– 13^. In specific therapeutic areas, like hypertension ^14^, mental illness ^15^, some cancers ^16^, HIV ^17^, and routine childhood vaccinations ^18^, biomedical research has generated enormous surplus economic value for the American public, far in excess of the sum of all public and private investments in research and development ^19^. These savings increase further when exclusivity ends, generics enter the market, and low-priced therapies become available to users of both the branded agent and other expensive medicines in the same class ^20^. Many new medicines also generate other valuable health and welfare benefits that are difficult to quantify, such as improving employment and social engagement for both patients and caregivers ^21^.

Public research and development investments have also been a significant growth driver for the U.S. economy and a wealth creator for taxpayers. This funding has yielded millions of relatively well-paid jobs and billions of dollars of taxes paid into the coffers of local communities, states, and the federal government ^22^, although these benefits take a long time to accrue and are often unevenly distributed across geographies.

An important caveat to these studies and conclusions is that just because publicly funded biomedical research yields large returns to taxpayers, does not mean that the current system for realizing those benefits is optimal. As discussed above, about 15 percent of new drugs are based on at least one patent that relied on public funding. In almost all cases, those patents were licensed to pharmaceutical firms under the Bayh-Dole Act, which was passed in 1980 to encourage the commercialization of taxpayer-funded research that might otherwise lie dormant ^23^. Since its passage, detractors have argued that academic patenting insufficiently rewards U.S. taxpayers for their contributions, and imposes costs that reduce its net social benefit ^24^. But even in light of possible opportunities to improve Bayh-Dole to create even more social value, the pharmaceutical industry is one of the clearest examples where exclusive intellectual property rights are critical to convert taxpayer-funded research into useful new products, because of the high cost and risk of drug development ^25^.

Calculating the net returns from publicly funded science is complicated, and it is unlikely that economists will ever explicitly quantify them in a way that satisfies all stakeholders. For this reason, it is impossible to determine objectively whether or not the extent of public support for drug development is appropriately accounted for in their prices. But this challenge notwithstanding, it is empirically false to argue that Americans “receive no return on the investment that they have made” in biomedical research.

## How is the “pay twice” claim relevant to the debate over high drug prices?

Recent proposals to limit drug prices are motivated by the worthy goal of ensuring that clinically valuable new drugs are not only developed, but also maximally available to the patients who need them ^26^. The pay twice critique has played an increasingly prominent role in justifying these legislative and administrative remedies, fueled by expensive medicines that owe at least some indisputable scientific debt to public research.

From a rhetorical standpoint, the pay twice argument certainly brings attention to the challenge of drug access and affordability. But practically speaking, prior experience suggests it would be difficult to link drug prices to the receipt of public support for basic biomedical research. NIH established a “reasonable pricing clause” in 1989 for products developed through some collaborative public-private research grants, which authorized the government to “require … reasonable evidence” of “a reasonable relationship between the pricing of a licensed product, the public investment in that product, and the health and safety needs of the public.” But the agency eliminated this provision in 1995, amidst concerns about how to define “reasonable pricing,” enforce restrictions and penalties, and mitigate potential negative effects on innovation ^27^. These concerns remain relevant today, in light of the persistent challenges outlined above in quantifying these factors, and would likely preclude adoption of a similar policy, especially if it were intended to apply to an even wider set of therapies.

Similarly, the feasibility of limiting drug prices via existing “march-in” rights is also limited. Bayh-Dole allows the government to obtain a nonexclusive, royalty-free license to patents developed with public funds, for its own use or that of a third party, if the patent holder fails to adequately commercialize the invention. The NIH has denied all of the march-in petitions it has received to date, maintaining that high prices per se are insufficient rationale to claim inadequate commercialization. ( A petition related to Exondys 51, a therapy for Duchenne muscular dystrophy, is still under review.) But even if this obstacle were surmounted, the practical impact of march-in rights would be limited, because almost all drugs covered by Bayh-Dole patents are also covered by additional privately held patents, to which the government has no claim ^28^.

Beyond the practical challenge of how to operationalize a link between a drug’s price and the extent to which public funds contributed to its development lies a more fundamental question: why should it matter? If one’s primary concern is that corporate profiteering limits affordable and equitable access to medicines with proven clinical benefits, then the pay twice argument undercuts the potential impact of broader proposals to improve access to all drugs, regardless of their provenance. Recent suggestions discussed by lawmakers and others include revoking monopoly pricing power once a certain profit threshold is exceeded; “delinking” patents from innovation and rewarding innovators instead with prizes; awarding the government more direct control over drug pricing via international reference price or cost-effectiveness benchmarks; and expanding government’s use of so-called “Section 1498” to use or manufacture any patented product ^29^. In parallel, proposals to eliminate out-of-pocket costs and ensure the availability of cheap generics after patent expiry could also improve access to drugs by reducing patient-borne expenses ^30^. These ideas all entail significant tradeoffs related to innovation that are incompletely understood, and reasonable stakeholders can disagree about how to weigh these tradeoffs given this inherent uncertainty ^31^. But importantly, they share a common focus on improving access to *all* clinically important therapies, irrespective of their origin, while ensuring that new drugs continue to be developed.

It is superficially attractive to argue that Americans are entitled to pay a lower price for a new drug that was substantially enabled by taxpayer-funded research. But the implication of that claim – that there is no such entitlement to affordability, or far less of one, for a drug mostly developed by a for-profit company – runs counter to the overarching goal of ensuring that all Americans have equitable access to beneficial therapies. Proposals to control a therapy’s price based on the degree to which public funds contributed to its development are not just unfeasible to implement, but also a distraction from more far-reaching efforts to improve the affordability of all medicines. Attention should instead be focused on developing practical solutions that ensure that clinically valuable new drugs continue to be developed and are accessible by all patients in need.

## Data availability

### Underlying data

No data are associated with this article.
