# Supplementary material for: Public research funding and pharmaceutical prices: do Americans pay twice for drugs?
Source: F1000Res. 2020 Jul 15;9:707. [Version 1] doi: 10.12688/f1000research.24934.1 (PMC7642989; doi:10.12688/f1000research.24934.1)
Supplement: Peer review history

# Peer review: Reviewer response for version 1

**DOI:** 10.5256/f1000research.27509.r73200

**Published:** 2020-11-04

**Authors:** Jacob Sherkow

**Affiliations:** 1 College of Law, University of Illinois at Urbana-Champaign, Champaign, USA

The authors succinctly and clearly explain why the "pay twice" critique regarding drug pricing in the U.S. is misguided using available evidence drawn from the literature. This is important because, as the authors note, critiques regarding "excessive" pricing "run[ ] counter to the overarching goal of ensuring that all Americans have equitable access to beneficial therapies." An excellent summary of a thorny—and unfortunately, persistent—topic in the literature.

Is the topic of the opinion article discussed accurately in the context of the current literature?

Yes

Are arguments sufficiently supported by evidence from the published literature?

Yes

Are all factual statements correct and adequately supported by citations?

Yes

Are the conclusions drawn balanced and justified on the basis of the presented arguments?

Yes

Reviewer Expertise:

intellectual property, patents, biotechnology, bioethics, regulation

I confirm that I have read this submission and believe that I have an appropriate level of expertise to confirm that it is of an acceptable scientific standard.

# Peer review: Reviewer response for version 1

**DOI:** 10.5256/f1000research.27509.r73012

**Published:** 2020-10-26

**Authors:** Fred D. Ledley

**Affiliations:** 1 Center for Integration of Science and Industry, Departments of Natural & Applied Science and Management, Bentley University, Waltham, MA, USA

This is a well written and referenced article and a significant contribution to the discussion about the relationship between public sector funding for biomedical research and drug prices by two accomplished scholars. We believe this is a meaningful contribution to the literature and should be published.

 We have several comments and suggestions for the authors and, since this is an open review, we offer these in the context of a more dialectical discussion than we would in a conventional review.

 As the author’s correctly note, there are two largely unrelated, issues in the “playing twice” policy debates:

1. Is the public actually paying for drug development while the company receives all of the profits based on the premise that they developed the drug?
2. Are taxpayers adequately rewarded for the contribution to new drug development? A corollary to this is the question of the role of Bayh-Dole in providing the public sector with a return.

We will address these arguments separately.

First, we would note that quote from Representative Ocasio-Cortez’ relates specifically to the second question, not the question of “paying twice”, and recommend that the author clarify this point.

 While the authors are absolutely correct in stating that “the vast majority of the credit for translating this research into new therapies is due to private companies,” we would point out that the framing of this question as relating solely to “translation” unfairly narrows the question. The problem is that, rhetorically framing the question as one that relates specifically to “translation’ or “development,” when the public sector contribution focuses primarily on basic biomedical research, makes their argument essentially a tautology.

 As the authors note, our research (referenced in the manuscript), demonstrates that >$100 billion in NIH funding was related to the drugs (or their targets) approved 2010-2016 www.pnas.org/content/115/10/2329, with >90% of this funding focused on basic research. We call the authors attention to a recent follow-on study, which shows that >$200 billion in NIH funding was related to the 356 drugs approved for the full decade 2010-2019. www.ineteconomics.org/uploads/papers/WP_133-Cleary-et-al-Govt-innovation.pdf, again, with the large majority focused on basic science.  If one assumes that industry spends an average of $1.5 billion in developing each drug, the amount estimated  by DiMasi *et al,* ^1^ pubmed.ncbi.nlm.nih.gov/26928437/ without considering cost of capital, (there is no cost of capital correction in calculating NIH spending), these data argue that the public sector contribution that enables drug discovery and development is of the same order of magnitude as the contribution of the private sector in the penultimate stages of translation. If one considers the range of drug development costs described by Wouters *et al* jamanetwork.com/journals/jama/fullarticle/2762311 ^2^, or the substantially lower estimates promulgated by some critics, the contributions of the public and private sectors may be very similar.

 The point is that BOTH the public sector investment in the enabling science AND the private sector investments in translation are required for drugs to reach the market, and that the overall investment needed to translate a scientific insight into an approved product is much greater than generally appreciated.  In this context, public DOES contribute a significant fraction of the overall cost of developing a drug, and DOES pay for the drug itself. Taking this value chain one step further, inasmuch as the revenues from sale of the drug provides the revenue for the large majority of industrial R&D, taxpayers are paying the FULL cost of both the basic research and the applied/translational research and development. Despite this, the public is ALSO paying for the profits of biopharmaceutical companies ( https://jamanetwork.com/journals/jama/fullarticle/2762308), and the price of drugs is, in fact, titrated to the desired profit rather than the cost of bringing drugs to market.  

 It is important to recognize that capital investments by shareholders contribute only a small fraction of the costs of research and development. The large pharmaceutical companies, which are primarily responsible for the costs of most drug development, distribute substantially more capital to shareholders in the form of dividends and stock buybacks than they raise in the form of stock sales www.ineteconomics.org/perspectives/blog/financialization-us-pharma-industry).

 Shareholders play a more significant role in funding R&D expenditures of emerging biotechnology companies. While capital investments in biotechnology companies have increased dramatically since 2015, annual investments in biotechnology companies are historically a small fraction of total R&D spending and not greater than public sector support through the NIH.

 We have several final suggestions for the authors (i) While we agree with the authors that taxpayers are not technically “paying twice,” we encourage the authors to acknowledge the limited frame of this question. (ii) We encourage the authors to recognize that the public sector is paying the large majority of the costs for R&D through taxes and drug prices, and ALSO subsidizing the distribution of cash to shareholders, who speculate in the success or failure of biopharmaceutical companies, but make little contribution to the development of new drugs.

Second, the argument that the public should receive a return on its investment in basic science, which was the basis of Representative Ocasio-Cortez’ question, arises from the work of Dr. Marianna Mazzucato (Dr. Mazzucato has consulted with Representative Ocasio-Cortez). As the authors reference, these arguments are articulated in her book The Entrepreneurial State (referenced in the article), and numerous articles in the academic literature. (e.g. hbr.org/2016/10/an-entrepreneurial-society-needs-an-entrepreneurial-state)  Her argument is that government serves as the “investor of first resort” in innovation, investing even before angel or venture investors, who expect high returns on their “risk investments.” Inasmuch as taxpayers are making even earlier investments with implicitly higher risk, Mazzucato argues that the public is similarly entitled to a proportionate return on investment. These arguments are increasingly important in public policy discussions and warrant further elaboration in the article. While the authors are technically correct in calling out the statement that taxpayers “receive no return” on investment as untrue, we would encourage them to refrain from rhetorically limiting the question to the issue of “NO” return.

 The authors correctly quote an extensive literature demonstrating the health value of effective medicines. We would note, however, that much of this work (particularly the series of landmark papers by Lichtenberg), largely reflect advances made in preventing and treating cardiovascular and infectious diseases, and there is increasing concern about the health value of many recently approved drugs, particularly those approved through expedited pathways. The authors also correctly note that job creation, taxes, and economic growth arising from pharmaceutical innovation constitute a public good and an indirect return on taxpayer investments. We would, however, encourage the authors to use more modest language than “…enormous surplus economic value…” in describing these benefits. We would also note that reference #19 does not appear to be relevant to this statement.

 We would, however, encourage the authors to recognize that these arguments are undercut by social inequities. The benefits of new drugs are not available to those without adequate insurance/government coverage due to their high price; the jobs created by the biopharmaceutical industry are disproportionately available in selected geographic areas and to individuals with access to higher education; companies go to extraordinary lengths to minimize the taxes they pay to the US government; and the profits generated by high drug prices are often distributed to shareholders who are disproportionately in the highest socioeconomic classes and who also go to great lengths to minimize their taxes. In this context, while the returns may not be as high as anticipated by policy makers due to tax avoidance strategies, and the aggregate or average benefits may not reflect the returns to most taxpayers.

 The authors should also recognize that the Bayh Dole Act captures a very small fraction of the value created by taxpayer investments in basic science and that the direct returns to the public sector are very limited. By design, Bayh-Dole is relevant only to “subject inventions” and the licenses that provide returns to nonprofit, academic organizations are predicated on patentable subject matter. The problem is that the legal definitions of an “invention” and patentable subject matter require that the inventor establish utility and the ability to reduce their advances to practice. In contrast, the primary focus of taxpayer-funded research is on basic science, which does not require recognized utility, though it may be “use inspired” (Stokes, Pasteur's Quadrant: Basic Science and Technological Innovation, Brookings, 1997) and is, by definition, not focused on enabling reduction to practice. Thus, the vast majority of government funded biomedical research is not covered by the Bayh-Dole Act and there is no formal mechanism for the public sector to receive a direct return on investment.

Is the topic of the opinion article discussed accurately in the context of the current literature?

Yes

Are arguments sufficiently supported by evidence from the published literature?

Yes

Are all factual statements correct and adequately supported by citations?

Yes

Are the conclusions drawn balanced and justified on the basis of the presented arguments?

Partly

Reviewer Expertise:

Characterizing systems for translational science including science, public and private sector investments, measures of public value creation, and public policy.

I confirm that I have read this submission and believe that I have an appropriate level of expertise to confirm that it is of an acceptable scientific standard.

## References

[1] : Innovation in the pharmaceutical industry: New estimates of R&D costs. *J Health Econ*.2016;47: 10.1016/j.jhealeco.2016.01.012 20-33 10.1016/j.jhealeco.2016.01.012 26928437

[2] : Estimated Research and Development Investment Needed to Bring a New Medicine to Market, 2009-2018. *JAMA*.2020;323(9) : 10.1001/jama.2020.1166 844-853 10.1001/jama.2020.1166 32125404PMC7054832

[3] : Profitability of Large Pharmaceutical Companies Compared With Other Large Public Companies. *JAMA*.2020;323(9) : 10.1001/jama.2020.0442 10.1001/jama.2020.0442 PMC705484332125401
